# Immunosuppressive mechanisms and therapeutic targeting of regulatory T cells in ovarian cancer

**DOI:** 10.3389/fimmu.2025.1631226

**Published:** 2025-07-09

**Authors:** Jing Li, Haojun Huang, Renxian Xie, Rongying Yang, Haitao Wang, Li Wan

**Affiliations:** ^1^ Department of Radiology, Shengjing Hospital of China Medical University, Shenyang, Liaoning, China; ^2^ School of Nursing, Yanbian University, Yanji, Jilin, China; ^3^ Department of Radiation Oncology, Cancer Hospital of Shantou University Medical College, Shantou, China; ^4^ Key Laboratory of Medical Electrophysiology, Ministry of Education and Medical Electrophysiological Key Laboratory of Sichuan Province, Institute of Cardiovascular Research, Southwest Medical University, Luzhou, Sichuan, China; ^5^ The School of Clinical Medical Sciences, Southwest Medical University, Luzhou, Sichuan, China; ^6^ Department of Neurosurgery, The First Hospital of China Medical University, Shenyang, Liaoning, China

**Keywords:** regulatory T cell, ovarian cancer, immunosuppression, metabolic reprogramming, immune checkpoint blockade

## Abstract

Ovarian cancer remains the most lethal gynecologic malignancy, largely due to its late-stage diagnosis and immunosuppressive tumor microenvironment (TME). A key mediator of immune evasion in ovarian cancer is the infiltration and activation of regulatory T cells (Tregs), which suppress antitumor immunity and foster therapeutic resistance. Emerging therapeutic strategies to target Tregs—such as cytokine modulation, checkpoint blockade, metabolic inhibitors, and epigenetic regulators—are critically evaluated for their potential to restore antitumor immunity. This review synthesizes recent advances in understanding how the ovarian TME shapes Treg biology, highlighting mechanisms such as cytokine signaling, chemokine-driven recruitment, metabolic reprogramming, and immune checkpoint interactions, as well as the phenotypic and functional heterogeneity of tumor-infiltrating Tregs, including tissue-resident and follicular subsets, and their clonal expansion in response to tumor antigens. By elucidating the dynamic crosstalk between Tregs and the ovarian TME, this review provides a framework for developing novel immunotherapies to overcome Treg-mediated immunosuppression and improve clinical outcomes.

## 1 Introduction

Ovarian cancer is one of the most prevalent malignant tumors affecting the female reproductive system (1). Due to its insidious onset and lack of early clinical symptoms, more than 70% of patients are diagnosed at an advanced stage, making it the leading cause of mortality among gynecologic malignancies (2). Investigating the origins and pathogenesis of ovarian cancer is therefore of paramount importance for developing effective strategies for its prevention and treatment. Accumulating evidence indicates that the onset of ovarian cancer is closely associated with impaired anti-tumor immunity in the host (3, 4). Furthermore, tumor immunosurveillance has been shown to correlate with clinical outcomes in ovarian cancer. Immune cells and the tumor immune microenvironment (TIME) actively participate in orchestrating the initiation and progression of ovarian malignancies.

Recent studies have demonstrated substantial immune cell infiltration within the ovarian tumor microenvironment, notably an enrichment of regulatory T cells (Tregs), a suppressive T cell subset. Increased Treg abundance has been strongly linked to immune evasion, poor prognosis, and elevated mortality risk in patients with ovarian cancer (5, 6). The differentiation and effector functions of tumor-infiltrating Tregs are governed by signals derived from the tumor microenvironment, playing a pivotal role in mediating therapeutic resistance and facilitating immune escape. This review aims to summarize recent advances in our understanding of how the ovarian cancer microenvironment regulates Treg cell biology, providing insights into their role in immune suppression and therapeutic resistance.

## 2 Regulatory T cells

### 2.1 Differentiation and effector mechanisms of Treg cells

Treg cells represent a subset of CD4^+^ T cells characterized by the expression of the lineage-defining transcription factor forkhead box protein P3 (FOXP3) (7). These cells exert potent immunosuppressive functions and play essential roles in maintaining immune homeostasis (8–10). They are critically involved in the regulation of immune tolerance and are implicated in the pathogenesis of autoimmune diseases and cancer (11–13). Based on their developmental origin, Treg cells can be broadly classified into thymus-derived regulatory T cells (tTregs) and peripherally induced regulatory T cells (iTregs) (9, 14, 15). tTregs originate from CD4^+^CD8^-^ thymocytes that exhibit high-affinity recognition of self-antigens (16). Upon T cell receptor (TCR) signaling and activation of the interleukin-2 (IL-2) pathway, FOXP3 expression is induced, conferring suppressive identity (17, 18). In contrast, iTregs differentiate from naive peripheral CD4^+^ T cells in response to TCR stimulation and exposure to cytokines such as IL-2 and transforming growth factor-β (TGF-β), which collectively induce FOXP3 expression (11, 19, 20). Functionally, Tregs mediate immune regulation by modulating immune tolerance and suppressing the activity of effector T cells, macrophages, dendritic cells, and B cells, primarily via immunosuppressive molecules and cytokines (21–25).

Treg cells express high levels of immunoregulatory surface molecules including cytotoxic T-lymphocyte-associated protein 4 (CTLA-4), CD39, and T cell immunoreceptor with immunoglobulin and immunoreceptor tyrosine-based inhibitory motif domain (TIGIT) (26–30). CTLA-4 on Tregs can bind the co-stimulatory molecules CD80 and CD86 on dendritic cells (DCs), thereby inhibiting antigen presentation (31, 32). In addition, CTLA-4 induces the expression of indoleamine 2,3-dioxygenase (IDO) in DCs, reducing tryptophan availability—a crucial nutrient for effector T cell proliferation and activation—thus dampening T cell responses (33). The ectoenzymes CD39 and CD73 on Tregs catalyze the hydrolysis of extracellular ATP to adenosine, a potent immunosuppressive metabolite that inhibits DC-mediated antigen presentation and T cell proliferation (34–37). TIGIT expression on Tregs is associated with their activation state and contributes to suppression by inducing the expression of fibrinogen-like protein 2 (Fgl2), which inhibits the activation of effector T cells and selectively attenuates inflammation mediated by T helper 1 (Th1) and Th17 cells (38). Tregs also exert suppressive functions through the secretion of cytokines such as TGF-β, IL-10, and IL-35, which inhibit both T cell and B cell effector functions (39). Upon activation, Tregs can secrete perforin and granzymes, leading to cytotoxic activity against effector CD4^+^ and CD8^+^ T cells (40). Moreover, as T cell proliferation and differentiation are IL-2-dependent processes, the high expression of CD25 on Tregs enables them to outcompete effector T cells for IL-2, resulting in cytokine deprivation that impairs effector T cell responses and contributes to immune suppression within the tumor microenvironment (41).

### 2.2 Treg cells in the ovarian cancer microenvironment

In patients with ovarian cancer, Treg cells are markedly enriched in both tumor tissues and ascites. These tumor-infiltrating Tregs (Ti-Tregs) can suppress anti-tumor effector responses and promote immune evasion, thereby facilitating tumor progression (42). Recent studies employing high-throughput sequencing have revealed distinct phenotypic and functional traits of Tregs within the ovarian tumor microenvironment. Laumont et al. (43) identified a subset of CD39^+^CD103^+^PD-1^+^ Tregs within tumors, displaying enhanced TCR diversity and a tissue-resident phenotype. Another study revealed that CEACAM1^+^ Tregs preferentially accumulate in tumor sites, are highly activated, and exhibit strong suppressive capacity. The accumulation of CEACAM1^+^ Tregs correlates with tumor progression; notably, their depletion enhances tumor-infiltrating lymphocyte (TIL) function and potentiates the therapeutic efficacy of anti-programmed death-1 (PD-1) therapy (44). TCR repertoire sequencing of Tregs in ovarian and other solid tumors has demonstrated considerable clonality and responsiveness to tumor-associated antigens, suggesting that intratumoral Tregs undergo clonal expansion and selection driven by antigen stimulation (45). Furthermore, a distinct subset of follicular regulatory T (Tfr) cells, which express chemokine receptor CXCR5, has been identified within the ovarian tumor microenvironment. Tfr cells, which typically reside in the germinal centers of secondary lymphoid tissues, regulate B cell responses. In ovarian cancer tissues and ascitic fluid, Tfr cells have been found to infiltrate and express high levels of TGFB1 and IL-10. Through IL-10 secretion, Tfr cells suppress the activation and cytotoxic function of CD8^+^ T cells, thereby contributing to an immunosuppressive microenvironment (46).

## 3 Mechanisms of Treg regulation by the ovarian cancer microenvironment

### 3.1 Cytokines regulate Treg biology

Tregs in ovarian cancer commonly express high levels of CD4, CD25, and FOXP3. Their increased presence is associated with immune evasion, lower survival rates, and elevated mortality risk in patients with ovarian cancer (6). Toker et al. (47), utilizing spatial transcriptomics, single-cell RNA sequencing, and TCR sequencing, identified the enrichment and heterogeneity of CD4^+^ Tregs in immune “cold” ovarian tumors. These findings suggest that Tregs constitute an immunosuppressive tumor microenvironment (TME) and are regulated by multiple factors.

**Figure 1:**
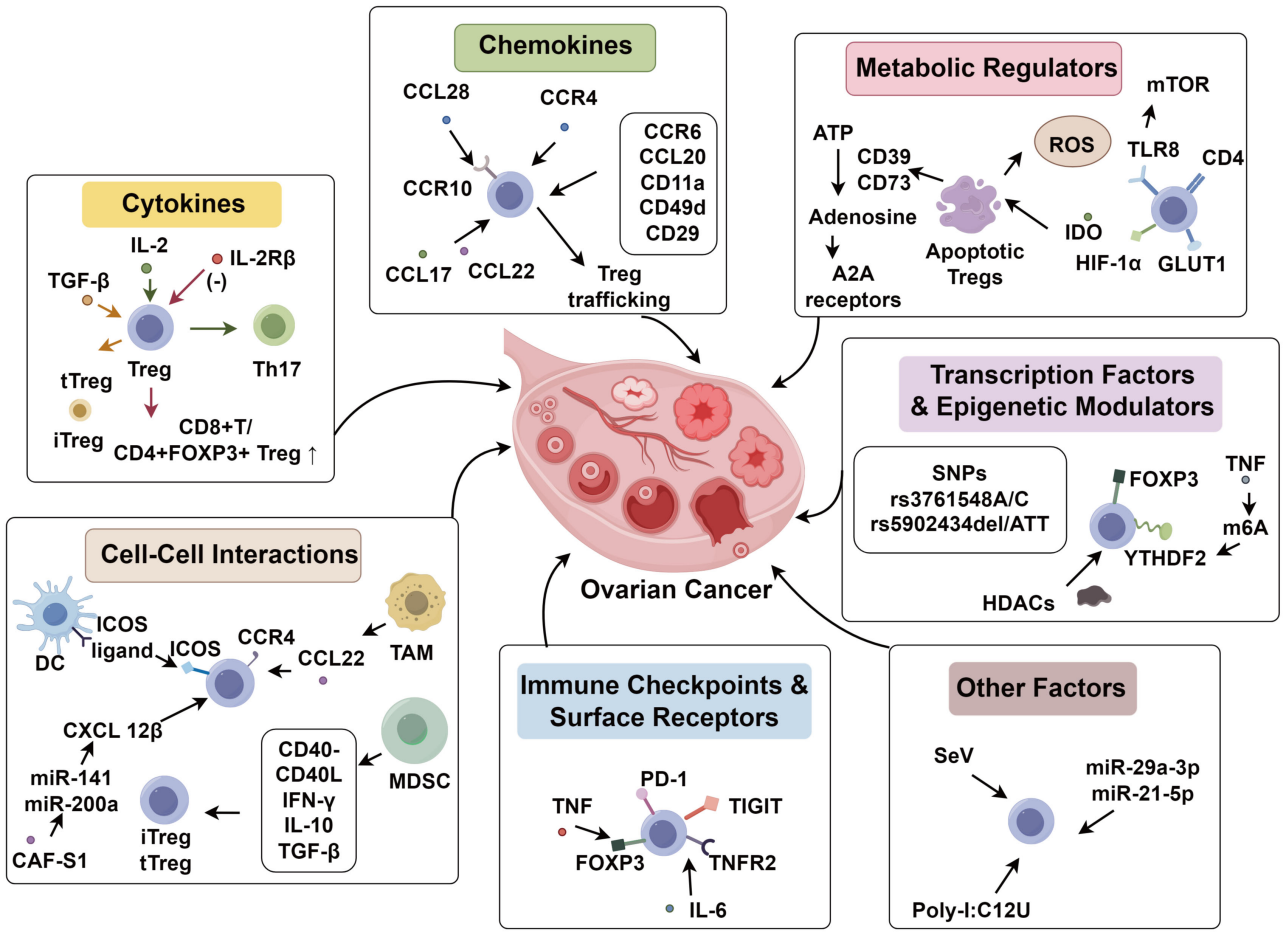
TME-mediated induction of immunosuppressive Tregs in ovarian cancer.

IL-2 signaling is essential for Treg development. Studies show ovarian tumor-derived CD4^+^CD25^+^ Tregs exhibit IL-2-dependent Th17 plasticity under CD3/APC stimulation, revealing microenvironmental modulation of Treg function (48). While high-affinity IL-2R inhibition reduces Tregs and tumor progression, it compromises effector T cells and induces autoimmunity. Drerup et al. (49) demonstrated low-affinity IL-2Rβ engagement increases Treg numbers while impairing suppression, improving CD8^+^/Treg ratios and tumor control, suggesting IL-2Rβ as a therapeutic target. TGF-β signaling similarly regulates Treg biology, with ovarian cancer cells secreting TGF-β to recruit tTregs and induce iTregs (50). TGF-β neutralization inhibits tumor progression, reduces ascites, and enhances CD8^+^/Treg ratios (51), while TGFBR2 SNPs further implicate this pathway in Treg modulation (52). Additional cytokines contribute to Treg regulation: M2-TAMs secrete TGF-α, IL-6, and IL-10 to maintain Tregs (53, 54), whereas Treg depletion enables IFN-α to stimulate DC-derived IL-6 and antitumor immunity (55) (**Figure 1**).

### 3.2 Chemokines mediate Treg trafficking and enrichment in the TME

Chemokines mediate Treg trafficking and enrichment in the TME. The CCL28 and CCL22/CCL17–CCR4 signaling axes selectively recruit Tregs into ovarian tumors (56, 57). In hypoxic *in vitro* ovarian cancer models, CCL28 expression is induced, promoting Treg migration via CCR10 engagement and suppressing host antitumor responses (56). Similarly, TAMs in ovarian tumors and ascites produce abundant CCL22, attracting Tregs and suppressing immune surveillance, whereas CCL22 inhibitors reduce T cell migration (57). Additional chemokines involved in Treg trafficking include CCR6/CCL20, CD11a, and integrins such as CD49d/CD29 (58, 59). Although CCR6 is highly expressed in hepatocellular and colorectal carcinomas and plays a role in autoimmune diseases (60), its contribution in ovarian cancer remains to be elucidated. Inhibition of the CXCR4–CXCL12 axis diminishes intratumoral Tregs and facilitates their conversion to helper T cells, enhancing intratumoral immune responses (61). Idorn et al. (62), using flow cytometry, revealed that FOXP3^-^ Treg infiltration in ovarian cancer correlates with CCR4 expression and enrichment of CCR4^+^, CCR5^+^, CXCR3^+^, and CXCR4^+^ T cells in both ascitic fluid and peripheral blood, linking this to elevated CCL22, CXCL9/10, and CXCL12 levels (**Figure 1**).

### 3.3 Immune checkpoints and surface receptors

Tregs in ovarian cancer highly express PD-1, which upon ligand binding suppresses immune activity. Tumor cells exploit this by upregulating PD-1 on Tregs to facilitate immune tolerance. PD-1 promotes apoptosis of inflammatory T cells while inhibiting apoptosis of Tregs, thereby increasing their proportion and contributing to immune suppression (47, 63). Sato et al. (64) found that CD45RA^-^FOXP3^+^ effector Tregs in ascites correlated with elevated PD-1 expression on CD8^+^ T cells. Compared to primary tumors, peritoneally metastasized ovarian cancers express higher PD-L1 levels, potentially enhancing Treg activation and promoting tumor progression (65). Tregs in ovarian cancer also overexpress TNFR2, a potent immunosuppressive receptor, likely in response to elevated IL-6 in the TME (66). High CCR4 expression in the TME facilitates TNFR2^+^ Treg accumulation in tumors and ascites (67). Active TNFR2 supports cell growth and modulates proliferation-apoptosis balance (68). TNF signaling enhances FOXP3 expression, maintaining the suppressive Treg phenotype (69). High CD73 expression in ovarian tumors correlates with poor prognosis. The CD73/adenosine axis supports the accumulation of Tregs and M2 macrophages, with Tregs suppressing antitumor immunity via CD73-dependent adenosine production (70). TIGIT, an inhibitory checkpoint molecule, is upregulated on CD4^+^ Tregs in murine ovarian cancer models. Anti-TIGIT antibody blockade reduces Treg numbers and suppressive function without affecting CD4^+^, CD8^+^, or NK cells, thereby improving survival (71). Recent clinical trials on ovarian cancer, including JAVELIN Ovarian 100 (NCT02718417) and JAVELIN Ovarian 200 (NCT02580058), demonstrated that the PD-L1 inhibitor avelumab, either as monotherapy or in combination with chemotherapy, did not significantly improve progression-free survival (PFS) or overall survival (OS) compared to chemotherapy alone (72, 73). Similarly, the KEYNOTE-100 trial evaluating pembrolizumab monotherapy reported a low objective response rate (ORR) (approximately 9.9%) and short duration of response (DOR) in patients with recurrent ovarian cancer (74). These findings suggest that immune checkpoint inhibitor monotherapy has limited efficacy in ovarian cancer, which may be attributed to Treg-mediated immunosuppression within the tumor microenvironment. These results underscore the need for rational combination strategies that concurrently target Tregs and stimulate effector immune responses to overcome the observed clinical resistance to immunotherapy (**Figure 1**).

### 3.4 Metabolic regulators

The aberrant metabolic milieu and metabolic byproducts in tumors influence Treg biology. Tregs in ovarian cancer tissues exhibit increased apoptosis, partially induced by adenosine, which also mediates immunosuppression. Maj et al. (36) found that apoptotic Tregs convert ATP to adenosine via CD39 and CD73, releasing adenosine that activates A2A receptors and dampens immune responses. Treg apoptosis is linked to their vulnerability to reactive oxygen species and diminished NRF2 antioxidant signaling, suggesting that oxidative stress-induced cell death enhances their immunosuppressive capacity. IDO also modulates Tregs by metabolizing tryptophan into kynurenine, which binds to aryl hydrocarbon receptors (AHRs) on T cells, skewing the Th17/Treg balance toward Tregs. Kynurenine also binds AHRs on TAMs, creating a feedback loop that upregulates IDO. Ovarian cancer progression is marked by a shift from Th17 dominance to Treg dominance, implicating metabolic byproducts in local immune modulation (75). Tryptophan deprivation induces AHR overexpression, promoting kynurenine uptake, AHR pathway activation, and Treg differentiation (76). Glucose metabolism also influences Treg function. Xu et al. (77) reported that Tregs in ovarian cancer overexpress glucose transporter 1 (GLUT1) and hypoxia-inducible factor 1-alpha (HIF-1α). TLR8 signaling suppresses mTOR activity, thereby modulating glycolysis and suppressing Treg functionality (**Figure 1**).

### 3.5 Transcription factors and epigenetic modulators

FOXP3 is the master transcription factor of Tregs. Studies on FOXP3 polymorphisms in ovarian cancer identified SNPs rs3761548A/C and rs5902434del/ATT as associated with epithelial ovarian tumor susceptibility and prognosis. The rs3761548A/C variant confers increased susceptibility, while rs5902434del/ATT is an independent prognostic factor (78). Epigenetic regulators also control Tregs. Class I histone deacetylases (HDACs) maintain Treg function; inhibition of HDACs suppresses Tregs, restores the CD8^+^/FOXP3^+^ Treg ratio, and reduces ascites (79). N6-methyladenosine (m6A), a key mRNA modification, regulates immunity via m6A reader proteins. TNF signaling induces expression of YTHDF2 in Tregs, which accelerates degradation of NF-κB repressors, thereby enhancing Treg activation and suppressive function. Conditional YTHDF2 deletion in Tregs impairs their function, increases apoptosis, and limits tumor growth, confirming the role of m6A regulation in tumor Tregs (80, 81) (**Figure 1**).

### 3.6 Cell-cell interactions

Tumor-associated DCs can promote Treg induction. Dysfunctional DCs in ovarian tumors facilitate Treg conversion (82). Conrad et al. (83) demonstrated that tumor-infiltrating Tregs overexpress ICOS, whose expansion depends on interactions with ICOS ligand on plasmacytoid DCs, promoting Treg proliferation and suppressive function. Additionally, DCs can induce IDO expression, facilitating Treg differentiation and T cell exhaustion (84). TAMs recruit CCR4^+^ Tregs via CCL22 secretion, suppressing T cell proliferation and enhancing immune evasion. Myeloid-derived suppressor cells (MDSCs) promote iTreg differentiation and tTreg expansion through CD40-CD40L, IFN-γ, IL-10, and TGF-β pathways (85–87). Cancer-associated fibroblasts (CAFs) also contribute to immune suppression. Givel et al. (88) found that the CAF-S1 subset in high-grade serous ovarian cancer (HGSOC) is enriched in mesenchymal tumors and promotes Treg chemotaxis, survival, and differentiation via a miR-141/miR-200a-dependent CXCL12β mechanism.

Theodoraki et al. (89) showed that TLR3 agonists such as Sendai virus (SeV), poly-I:C, and rintatolimod (poly-I:C12U) activate IFN-α and CXCL10 expression, enhancing T cell infiltration. These agonists also stimulate MAVS signaling, inhibiting NF-κB and TNF-α-dependent COX2 activation. The COX2/PGE2 pathway promotes Treg infiltration by inducing IDO, IL-10, CCL22, and CXCL12. Extracellular vesicles also regulate Tregs in ovarian cancer. TAM-derived exosomes enriched in miR-29a-3p and miR-21-5p inhibit STAT3 in CD4^+^ T cells, promoting Treg differentiation and expression of TGF-β and IL-10, while suppressing Th17 differentiation and TNF-α/IL-6 secretion (90) (**Figure 1**).

## 4 Ovarian cancer therapy targeting Treg in the TME

### 4.1 Inhibiting Treg proliferation and recruitment

Given the pivotal immunosuppressive role of Tregs in ovarian cancer, targeted strategies to disrupt their function have emerged as promising therapeutic approaches. While IL-2 demonstrates clinical efficacy with response rates of 16-20% in cancer patients, its therapeutic potential is limited by the paradoxical expansion of CD4^+^CD25^+^FOXP3^+^ Tregs (91). To overcome this limitation, novel agents have been developed, including Ontak, a fusion protein of IL-2 and diphtheria toxin, which selectively depletes Tregs through inhibition of protein synthesis, and nemvaleukin alfa, an engineered cytokine that preferentially activates CD8^+^ T cells and NK cells while minimizing Treg expansion (NCT02799095) (92, 93). Alternative strategies focus on blocking key Treg pathways, such as CTLA-4 inhibition with MDX-CTLA-4 which reduces CA125 levels, or CCR4 targeting with mAb2–3 that stimulates IFN-γ while suppressing IL-2-driven Treg proliferation (94, 95). Additionally, immunotherapeutic approaches like the GVAX whole-cell vaccine enhance antitumor immunity through GM-CSF-mediated CTL infiltration and Treg reduction, demonstrating the potential of combinatorial strategies to effectively modulate the immunosuppressive tumor microenvironment in ovarian cancer (96).

### 4.2 Modulate Treg biosynthesis, differentiation, or suppressive function

Cyclophosphamide, an alkylating agent that disrupts DNA Treatment with MDX-CTLA-4 in ovarian cancer patients replication in rapidly dividing cells, preferentially eliminates highly proliferative Tregs in the tumor milieu at low doses, thereby potentiating antitumor immunity (97). Combined use of cyclophosphamide with intratumoral Bacillus Calmette–Guérin (BCG) vaccination has been shown to diminish Treg frequencies while enhancing CD8^+^ T cell infiltration (98). Other compounds, including mitoxantrone, the glycolytic inhibitor 2-deoxy-D-glucose (2-DG), and IDO inhibitors, similarly reduce Treg numbers and impair their immunosuppressive activity (99–101). Recent findings indicate that anionic liposomal delivery of Toll-like receptor (TLR) antagonists in ovarian cancer leads to a reduction in Treg accumulation, concurrent with elevated T cell infiltration and M1 macrophage polarization within the TME (102).

### 4.3 Inhibition of tumor angiogenesis

Another therapeutic axis centers on the inhibition of tumor angiogenesis. Vascular endothelial growth factor (VEGF), often overexpressed in the TME, has been implicated in the recruitment of Tregs. Application of anti-VEGF monoclonal antibodies in ovarian cancer has been associated with a reduction in circulating Tregs and a concomitant rise in effector T cell populations (103). Programmed death-1 (PD-1), an immunoinhibitory receptor, facilitates immune escape in tumors (104). OX40, a co-stimulatory receptor expressed on activated T cells and Tregs, belongs to the tumor necrosis factor receptor superfamily and plays a crucial role in T cell activation and expansion. Notably, combinatorial blockade of PD-1 and activation of OX40 signaling induces a robust immunostimulatory response in ovarian cancer murine models, characterized by elevated IFN-γ levels and diminished Treg infiltration (105). Furthermore, CCR4 is highly expressed on Tregs, and chemokines secreted by tumor cells can attract CCR4^+^ Tregs into the tumor niche (106). Consequently, targeting CCR4 in clinical trials holds potential to provide a new and reliable strategy for immunotherapeutic intervention in ovarian cancer (**Table 1**).

**Table 1:** Therapeutic strategies targeting Tregs in ovarian cancer.

Therapeutic approach	Target	Effects on Tregs/TME	Results
Cytokine Modulation	IL-2Rβ agonists (low-affinity)	Reduces Treg suppression while sparing CD8^+^ T cells.	Improves CD8^+^/Treg ratio; delays tumor growth in models.
Checkpoint Inhibition	Anti-CTLA-4 (e.g., MDX-CTLA-4)	Depletes Tregs via ADCC; enhances Teff activity.	Lowers CA125 levels in patients.
Chemokine Axis Blockade	Anti-CCR4 (mAb2-3)	Inhibits Treg recruitment; boosts IFN-γ.	Synergizes with PD-1 blockade.
Metabolic Interference	IDO inhibitors (e.g., epacadostat)	Reverses kynurenine-mediated Treg polarization.	Restores Th17/Treg balance; trials show mixed efficacy.
Epigenetic Modulation	HDAC inhibitors (entinostat)	Reduces FOXP3^+^ Treg stability; increases CD8^+^/Treg ratio.	Decreases ascites and tumor burden in preclinical models.
Combination Therapies	Cyclophosphamide + BCG vaccine	Selectively depletes proliferating Tregs; enhances CD8^+^ infiltration.	Improves response to immunotherapy.
Anti-Angiogenics	Bevacizumab (anti-VEGF)	Reduces Treg recruitment; normalizes vasculature.	Transiently elevates Teff populations.
TLR Agonists	Poly-I:C (TLR3 agonist)	Induces IFN-α/CXCL10; suppresses COX2/PGE2-driven Treg recruitment.	Enhances T cell infiltration; phase I/II trials ongoing.

## 5 Conclusion

Ovarian cancer remains a formidable challenge in oncology, with its immunosuppressive tumor microenvironment playing a critical role in disease progression and therapeutic resistance. Regulatory T cells are key mediators of immune evasion, suppressing antitumor responses through multiple mechanisms, including cytokine secretion, metabolic regulation, and immune checkpoint interactions. Despite advances in immunotherapy, clinical trials targeting PD-1/PD-L1 in ovarian cancer have shown limited efficacy, underscoring the need for novel strategies that disrupt Treg-mediated immunosuppression. Emerging approaches such as Treg depletion, inhibition of recruitment signals, metabolic modulation, and combination therapies targeting immune checkpoints hold promise for restoring antitumor immunity. Future research should focus on identifying biomarkers for patient stratification and optimizing combinatorial regimens that simultaneously neutralize Treg suppression while enhancing effector T cell function. Overcoming Treg-driven resistance will be crucial for improving outcomes in ovarian cancer immunotherapy.
